# Messenger RNA Expression of Selected Factors at Different Sites of the Bovine Endometrium Associated With Uterine Health

**DOI:** 10.3389/fvets.2021.649758

**Published:** 2021-03-05

**Authors:** Harald Pothmann, Paula Flick, Alexander Tichy, Christoph Gabler, Marc Drillich

**Affiliations:** ^1^Clinical Unit for Herd Health Management in Ruminants, University Clinic for Ruminants, Vetmeduni Vienna, Vienna, Austria; ^2^Department of Veterinary Medicine, Institute of Veterinary Biochemistry, Freie Universität Berlin, Berlin, Germany; ^3^Department of Scientific Biomedicine, Platform of Bioinformatics and Biostatistics, Vetmeduni Vienna, Vienna, Austria

**Keywords:** mRNA, pro-inflammatory factors, uterine health, endometrium, cows

## Abstract

Recent studies have elucidated the role of several pro-inflammatory factors as mediators of inflammatory processes in the bovine endometrium. Only few studies, however, have analyzed samples collected from different regions of the uterus of the same animal. In this study, we tested the hypothesis that on a molecular level, clinical endometritis is characterized by inflammatory responses spread over the entire endometrium. Furthermore, we assume that subclinical endometritis is described by an inflammation of local regions of the uterus. Therefore, the objective of this study was to assess the mRNA expression of uterus-associated pro-inflammatory factors at five pre-defined endometrial sites, i.e., *corpus uteri*, left horn base, left horn tip, right horn base, and right horn tip, in cows with clinical and subclinical endometritis and in healthy controls. We analyzed the mRNA expression of interleukin 1 alpha, interleukin 1 beta, C-X-C motif chemokine ligand 8, prostaglandin-endoperoxide synthase 2, protein tyrosine phosphatase receptor type C, carcinoembryonic antigen related cell adhesion molecule 1, and mucin 4 and 16. Based on vaginoscopy and endometrial cytology (≥ 5% polymorphonuclear neutrophils) between 28 to 34 days in milk, 18 Simmental cows were categorized in clinical endometritis group (*n* = 7), subclinical endometritis group (*n* = 4), and healthy group (*n* = 7). In general, the analyses revealed a great variation of mRNA expression between sites and animals. Differences were found between different uterine health statuses, but the variation between the sampling sites within the groups was not significant (*P* > 0.05). This indicates that inflammatory processes at the end of the postpartum period can be regarded as multi-focal or spread throughout the uterus independent from the uterine health status.

## Introduction

Postpartum (pp) uterine disorders, such as metritis, clinical endometritis (CE), and subclinical endometritis (SE), have negative short- and long-term impact on bovine fertility (1–3). Bacterial infections provoke the release of pro-inflammatory factors, such as prostaglandins and cytokines, e.g., interleukin 1A (*IL1A*), interleukin 1 B (*IL1B*), and C-X-C motif chemokine ligand 8 (*CXCL8*) (4–6). These chemoattractants play a central role in the innate immune response to regulate the migration of immune cells, e.g., polymorphonuclear neutrophils (PMN), which accomplish phagocytosis in the uterine tissue (7). Detailed information about the physiological function of pro-inflammatory factors involved in inflammatory processes was recently reviewed by Pascottini et al. (8).

The cytobrush (CB) technique was reported as a reliable and accurate method for endometrial sampling and the diagnosis of SE by determining the proportion of PMN in the smears (9–11). It has been discussed whether a single sample is representative for the entire endometrium and affects the accuracy of the diagnosis of SE [reviewed by (12, 13)]. The CB technique can also be used to evaluate the mRNA expression in the endometrial samples. An increased mRNA expression of cytokines was found in cows with SE and CE compared with healthy controls (14, 15). Repeated sampling of the same cows showed no or only few intra-cow variations of the mRNA expression of selected factors (16, 17).

The aim of the present study was to assess mRNA pattern of transcripts involved in physiological and pathological processes, obtained from different endometrial sites *in vivo* in cows with different uterine health status. In this study, we tested the hypothesis that on a molecular level, clinical endometritis is characterized by inflammatory responses spread over the entire endometrium. Furthermore, we assume that subclinical endometritis is characterized by an inflammation of local regions of the uterus. As additional comparison group, we included healthy control cows.

## Materials and Methods

### Study Design

This study was approved by the institutional ethics committee and the national authority according to §8 of Law for Animal Experiments, Tierversuchsgesetz-TVG (BMWFW-68.205/0156-WF/II/3b/2014) and performed at the research farm VetFarm Kremesberg, University of Veterinary Medicine Vienna, Austria.

The herd comprised ~80 lactating dairy cows, housed in free stall barns with cubicles. Eighteen Simmental cows (14 multipara, four primipara, no history of Cesarean section or dystocia) were enrolled. All cows were examined between day 28 to 34 pp by transrectal palpation and vaginoscopy, followed by uterine CB sampling at five pre-defined sites. Cows with purulent or mucopurulent vaginal discharge were defined as affected with CE (18). Subclinical endometritis was characterized by the absence of vaginal discharge, but with ≥ 5% PMN in one of the five endometrial smears (19). The absence of vaginal discharge and a proportion of <5% PMN in the cytological samples defined the healthy control (HC) cows.

### Endometrial Sampling

Five endometrial samples were collected by CB technique from each cow in the same order of sampling, as described (13). In brief, the CB (Gynobrush, Heinz Herenz, Hamburg, Germany) was inserted through a metal tube (50 cm in length, 6 mm inner diameter) and forwarded under manual control to the sites in the following order: (i) *corpus uteri* (CU), (ii) base of the left horn (LHB), (iii) tip of the left horn (LHT), (iv) base of the right horn (RHB), and (v) tip of the right horn (RHT). After retraction, the CB was rolled onto a microscope slide and thereafter stored in tubes at −80°C for RNA analysis. The slides were dried, fixed, stained (Hemacolor Merck, Darmstadt, Germany) and examined under a microscope by using X 400 magnification (Olympus CX21, Olympus, Tokyo, Japan). The percentage of PMN was determined by counting 300 endometrial cells and PMN in total (11).

### Total RNA Extraction, Reverse Transcription, and Real-Time PCR

After thawing the CB, total RNA was extracted using RNeasy Plus Mini Kit (Qiagen, Hilden, Germany) according to the manufacturer's instructions. Quantification of total RNA was performed by spectrophotometry (NanoDrop ND-1000 Peqlab Biotechnology, Erlangen, Germany) at 260 nm. Quality and integrity of the isolated total RNA was assessed using the Bioanalyzer 2100 and the Agilent RNA 6000 Nano Kit (both Agilent Technologies, Waldbronn, Germany). The RNA integrity number of the isolated RNA was greater 8.0 and the OD 260/280 > 2.0. Possible genomic DNA contaminations were removed by using DNase. Reverse transcription to generate DNA was performed as described (16). In brief, single stranded cDNA was generated from 100 ng total RNA per sample with the addition of 200 U RevertAid Reverse Transcriptase and 2.5 μM random hexamer primers (both Thermo Scientific, Schwerte, Germany) in a total volume of 60 μl. To confirm the absence of any genomic DNA or contaminations, samples without reverse transcriptase were prepared as negative controls. The generated DNA served as template for real-time PCR to quantify mRNA of the selected factors in the endometrial cells. Gene transcripts, primer sequences and further parameter used for real-time PCR are listed in Supplementary Table 1.

Real-time PCR was conducted using the Rotor-Gene 3000 (Corbett Research, Mortlake, Australia), as described (16). In brief, amplification of 1 μl cDNA per sample was carried out in the presence of 0.4 μM of each primer (forward and reverse) and 5 μl 2 × SensiMix SYBR Low-ROX (Bioline, Luckenwalde, Germany) in a total reaction volume of 10 μl. Denaturation at 95°C for 10 min was followed by a three-step amplification in 45 cycles: denaturation at 95°C for 15 s, annealing for 20 s (Supplementary Table 1), and extension at 72°C for 30 s. Subsequently, a melting curve program (50–99°C) with continuous fluorescence measurement confirmed specific amplification.

Quantification of mRNA was performed with dilution series and comparison with standards amplified simultaneously. Messenger RNA expression of each sample was normalized with the succinate dehydrogenase complex flavoprotein subunit A (*SDHA*) and glyceraldehyde-3-phosphate dehydrogenase (*GAPDH*) real-time PCR data. Calculation of mRNA expression of protein tyrosine phosphatase receptor type C (*PTPRC*; formerly known as *CD45*) and carcinoembryonic antigen related cell adhesion molecule 1 (*CEACAM1*; formerly known as CD66a*)*, prostaglandin-endoperoxide synthase 2 (*PTGS2*), *IL1A, IL1B*, C-X-C motif chemokine ligand 8 (C-X-C motif) ligand 8 (*CXCL8*; formerly known as *IL8*), and mucins (*MUC*) MUC4 and *MUC16* was performed using Rotor-Gene 6.1 software.

### Statistical Analysis

Statistical analyses were performed with SPSS Statistics version 23.0 (IBM, New York, USA). Data was tested for normality with Kolmogorov-Smirnov test. For each sample, normalized mRNA expression for the selected factors at the five pre-defined endometrial sites was calculated. Median, interquartile range (IQR), mean and standard deviation (SD) of normalized mRNA expression were calculated to describe the variation within cows with different uterine health status. At the level of endometrial sites, the variance of normalized mRNA expression, determined as the coefficient of variation (CV: quotient of SD and mean) was calculated. The normally distributed data was analyzed by ANOVA using the CV as dependent variable and HC, SE, and CE as factors to compare with. Scheffe test was conducted for *post hoc* multiple comparisons. Normalized mRNA expression of selected transcripts on cow-level, categorized according to uterine health status, was shown in box plots and compared by Kruskal-Wallis test and pairwise by Mann-Whitney-*U* test. The level of significance was set at *P* < 0.05.

## Results

**Figure 1:**
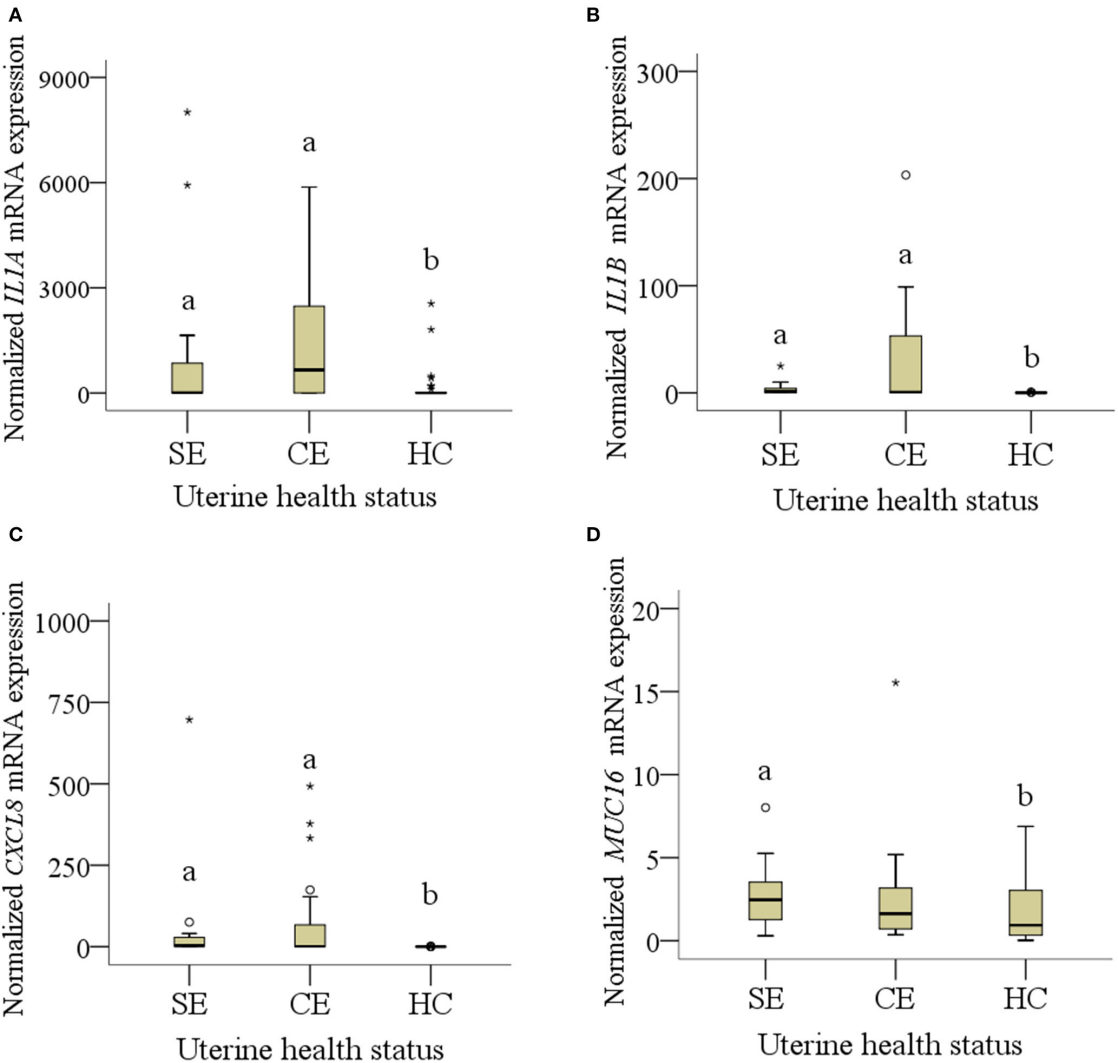
**(A–D)**: mRNA expression of normalized **(A)** *IL1A*, **(B)** *IL1B*, **(C)** *CXCL8* and **(D)** *MUC16* collected from four cows (20 samples) with subclinical endometritis (SE), seven cows (31 samples*) with clinical endometritis (CE) and seven (35 samples) healthy cows (HE). In CE, four samples were missing because of failed cytobrush sampling. Only diagrams showing significant results of pro-inflammatory factors and mucins are presented. Different letters indicate significant differences between groups: a:b < 0.05. The circle mean outliers and asterisks mean extreme outliers (values).

Seven cows were diagnosed with CE, four with SE, and seven were regarded as healthy (HC). Descriptive statistics and statistical analyses of mRNA expression at the five different endometrial sites related to uterine health (CE, SE, HC) are presented in Tables 1–3. Proportion of normalized mRNA expression of selected pro-inflammatory factors from cows with different uterine health status (SE, CE, and HC) and significant differences are shown in Figure 1.

**Table 1:** Median, interquartile range (IQR), mean, and standard deviation (SD) of normalized mRNA expression for different pro-inflammatory factors and mucins in the endometrium at the *corpus uteri* (CU), the base of the left horn (LHB), the tip of the left horn (LHT), the base of the right horn (RHB) and the tip of the right horn (RHT) collected from seven cows with clinical endometritis (CE) on day 28 to 34 postpartum.

**CE cows (*****n*** **= 7)**		**Endometrial sites**				
**Factor**		**LHT**	**RHT**	**RHB**	**LHB**	**CU**
PTGS2	Median (IQR)	1.6 (50.1)	0.5 (209.6)	1.6 (331.6)	3.2 (21.9)	1.1 (16.6)
	Mean (SD)	177.8 (437.9)	84.0 (178.3)	132.9 (265.0)	111.4 (281.7)	205.4 (535.2)
PTPRC	Median (IQR)	0.7 (2.5)	1.0 (0.8)	1.5 (5.8)	0.6 (1.7)	0.6 (1.7)
	Mean (SD)	1.5 (1.5)	0.6 (1.5)	2.8 (3.9)	1.1 (1.2)	1.7 (2.6)
CEACAM1	Median (IQR)	3.3 (2.8)	2.5 (9.7)	1.5 (9.4)	1.2 (4.2)	1.6 (6.1)
	Mean (SD)	2.6 (5.0)	4.5 (6.5)	4.7 (6.3)	5.5 (10.8)	5.1 (8.9)
Il1A	Median (IQR)	659.9 (2,237)	608.5 (26,878)	1,815 (19,737)	228.4 (3,865)	1,841 (5,871)
	Mean (SD)	36,761 (95,367)	10,872 (23,531)	8,296 (15,950)	13,039 (31,750)	26,511 (65,930)
Il1B	Median (IQR)	0.8 (86.3)	0.3 (147.9)	1.1 (36.0)	0.5 (40.1)	0.6 (98.8)
	Mean (SD)	242.0 (578.9)	59.2 (89.9)	14.7 (25.3)	13.8 (22.7)	182.9 (440.3)
CXCL8	Median (IQR)	1.6 (377.5)	0.8 (283.0)	1.1 (171.9)	2.0 (59.6)	0.7 (6.3)
	Mean (SD)	476.2 (1,034)	113.4 (214.3)	69.1 (147.8)	34.7 (65.3)	590.0 (1,557)
MUC4	Median (IQR)	41.6 (107.8)	27.4 (279.0)	35.5 (52.5)	3.3 (101.5)	47.6 (138.7)
	Mean (SD)	54.4 (50.1)	118.0 (180.2)	29.6 (26.9)	36.5 (55.6)	122.0 (203.7)
MUC16	Median (IQR)	2.3 (2.5)	1.5(2.7)	0.8 (7.9)	0.9 (2.1)	3.2 (2.8)
	Mean (SD)	2.3 (1.6)	1.7 (1.6)	3.8(5.6)	1.8 (1.7)	2.5 (1.3)

*PTGS2, prostaglandin-endoperoxide synthase 2; PTPRC, protein tyrosine phosphatase receptor type C; CEACAM1, carcinoembryonic antigen related cell adhesion molecule 1; IL1A, interleukin 1 alpha; IL1B, interleukin 1 beta; CXCL8, C-X-C motif chemokine ligand 8; MUC4, mucin 4; MUC16, mucin 16*.

**Table 2:** Median, interquartile range (IQR), mean, and standard deviation (SD) of normalized mRNA expression for different pro-inflammatory factors and mucins in the endometrium at the *corpus uteri* (CU), the base of the left horn (LHB), the tip of the left horn (LHT), the base of the right horn (RHB) and the tip of the right horn (RHT) collected from four cows with subclinical endometritis (SE) on day 28 to 34 postpartum.

**SE cows (*****n*** **= 4)**		**Endometrial sites**				
**Factor**		**LHT**	**RHT**	**RHB**	**LHB**	**CU**
PTGS2	Median (IQR)	19.2 (141.0)	3.9 (3.4)	25.2 (56.8)	80.2 (554.0)	27.3 (417.9)
	Mean (SD)	56.0 (85.9)	4.0 (1.8)	28.7 (30.0)	212.9 (326.0)	149.4 (262.6)
PTPRC	Median (IQR)	0.6 (1.9)	2.1 (3.4)	0.4 (1.8)	0.7 (0.4)	0.9 (1.9)
	Mean (SD)	1.2 (1.2)	2.1 (1.8)	1.0 (1.1)	0.7 (0.2)	1.1 (1.0)
CEACAM1	Median (IQR)	3.3 (16.4)	2.8 (8.1)	0.5 (16.2)	3.6 (24.7)	2.3 (21.0)
	Mean (SD)	6.9 (9.5)	3.9 (4.4)	5.7 (10.6)	9.7 (14.8)	7.9 (12.9)
Il1A	Median (IQR)	826.0 (6,414)	1.8 (5.3)	12.8 (4,446)	7.9 (14,777)	34.6 (17,190)
	Mean (SD)	2,415 (3,808)	2.4 (2.9)	1,489 (2,959)	4,928 (9,846)	5,748 (11,444)
Il1B	Median (IQR)	1.6 (.2)	0.3 (1.6)	1.3 (4.1)	1.8 (8.2)	5.9 (20.0)
	Mean (SD)	3.3(4.7)	0.6 (0.9)	2.0 (2.2)	3.5 (4.7)	9.4 (11.1)
CXCL8	Median (IQR)	3.2 (28.6)	2.5 (56.7)	4.0 (31.0)	15.8 (27.7)	6.2 (524.1)
	Mean (SD)	11.3 (17.9)	20.2 (36.8)	12.2 (19.0)	15.1 (15.6)	177.6 (346.3)
MUC4	Median (IQR)	6.1 (43.2)	98.0 (243.9)	9.6 (70.3)	12.4 (60.2)	9.2 (631.1)
	Mean (SD)	17.1 (25.8)	114.7 (134.0)	26.8 (41.6)	24.9 (34.4)	213.7 (415.0)
MUC16	Median (IQR)	2.8 (3.5)	1.2 (1.6)	2.4 (5.4)	3.3 (1.8)	2.4 (2.2)
	Mean (SD)	3.0 (1.8)	1.3 (0.9)	3.5 (3.1)	3.5 (0.9)	2.5 (1.1)

*PTGS2, prostaglandin-endoperoxide synthase 2; PTPRC, protein tyrosine phosphatase receptor type C; CEACAM1, carcinoembryonic antigen related cell adhesion molecule 1; IL1A, interleukin 1 alpha; IL1B, interleukin 1 beta; CXCL8, C-X-C motif chemokine ligand 8; MUC4, mucin 4; MUC16, mucin 16*.

**Table 3:** Median, interquartile range (IQR), mean, and standard deviation (SD) of normalized mRNA expression for different pro-inflammatory factors and mucins in the endometrium at the *corpus uteri* (CU), the base of the left horn (LHB), the tip of the left horn (LHT), the base of the right horn (RHB) and the tip of the right horn (RHT) collected from seven healthy cows (HC) on day 28 to 34 postpartum.

**HC cows (*****n*** **= 7)**		**Endometrial sites**				
**Factor**		**LHT**	**RHT**	**RHB**	**LHB**	**CU**
PTGS2	Median (IQR)	6.1 (10.2)	3.1 (5.3)	7.0 (8.1)	9.2 (22.2)	4.6 (17.2)
	Mean (SD)	33.3 (76.0)	3.6 (3.4)	7.2 (8.1)	39.1 (78.0)	17.7 (29.9)
PTPRC	Median (IQR)	0.5 (0.8)	0.6 (1.4)	0.5 (0.2)	0.6 (0.5)	0.5 (0.4)
	Mean (SD)	0.8 (0.6)	0.9 (0.6)	0.5 (0.5)	0.7 (0.8)	0.3 (0.5)
CEACAM1	Median (IQR)	4.0 (11.8)	2.5 (4.2)	2.7 (13.6)	3.1 (35.8)	4.1 (10.0)
	Mean (SD)	5.7 (6.4)	4.0 (5.0)	6.5 (9.0)	13.7 (17.3)	6.0 (5.4)
Il1A	Median (IQR)	0.01 (1.9)	0.4 (22.4)	5.6 (206.4)	0.3 (203.3)	0.2 (0.6)
	Mean (SD)	258.8 (683.8)	64.8 (160.4)	410.5 (946.9)	98.6 (185.3)	26.9 (70.6)
Il1B	Median (IQR)	0.1 (0.2)	0.1 (0.6)	0.1 (0.2)	0.1 (0.2)	0.01 (0.1)
	Mean (SD)	0.2 (0.2)	0.4 (0.6)	0.1 (0.2)	0.1 (0.1)	0.1 (0.1)
CXCL8	Median (IQR)	0.1 (0.2)	0.01 (0.8)	0.1 (0.2)	0.1 (0.2)	0.1 (0.2)
	Mean (SD)	0.2 (0.3)	0.7 (1.3)	0.6 (1.3)	0.2 (0.3)	0.1 (0.2)
MUC4	Median (IQR)	6.5 (23.4)	11.2 (130.0)	1.2 (30.8)	15.0 (21.5)	6.6 (20.2)
	Mean (SD)	19.2 (30.4)	68.1 (90.1)	31.9 (66.3)	18.2 (25.0)	24.8 (49.3)
MUC16	Median (IQR)	1.0 (5.7)	0.9 (2.3)	0.8 (1.3)	0.9 (3.5)	2.8 (4.6)
	Mean (SD)	2.3 (2.8)	1.3 (1.2)	1.3 (1.7)	1.7 (2.1)	2.2 (2.4)

*PTGS2, prostaglandin-endoperoxide synthase 2; PTPRC, protein tyrosine phosphatase receptor type C; CEACAM1, carcinoembryonic antigen related cell adhesion molecule 1; IL1A, interleukin 1 alpha; IL1B, interleukin 1 beta; CXCL8, C-X-C motif chemokine ligand 8; MUC4, mucin 4; MUC16, mucin 16*.

### Endometrial mRNA Expression at the Five Sampling Sites

Comparing the median mRNA expression at the five pre-defined sites resulted in no differences within cows with SE, CE, and HC cows (Kruskal-Wallis test: *P* > 0.05, Tables 1–3). Overall, the high IQR indicated a great variation of mRNA expression at all different endometrial sites, in particular in cows with CE (Tables 1–3). Moreover, the CV of mRNA expression at the five endometrial sites was high and revealed no differences between the groups CE, SE, and HC (Table 4).

**Table 4:** Oneway ANOVA of the coefficient of variation (CV) for the normalized mRNA expression of selected transcripts in five pre-defined endometrial sites obtained from seven cows with clinical endometritis (CE), four with subclinical endometritis (SE), and seven healthy control cows (HC) on days 28 to 34 postpartum.

**Factor**	**Group**	**Mean**	**SD**	**SEM**	**CI lower bound**	**CI upper bound**	***P***
PTGS2	CE	1.2	0.6	0.2	0.659	1.827	0.26
	SE	0.94	0.2	0.1	0.693	1.192	
	HC	0.7	0.4	0.1	0.417	1.109	
PTPRC	CE	0.8	0.5	0.2	0.372	1.197	0.83
	SE	0.8	0.4	0.2	0.107	1.452	
	HC	0.6	0.2	0.1	0.431	0.867	
CEACAM1	CE	0.7	0.3	0.1	0.454	1.042	0.73
	SE	0.6	0.2	0.1	0.263	0.972	
	HC	0.7	0.2	0.1	0.475	0.937	
IL1A	CE	1.1	0.6	0.2	0.552	1.670	0.82
	SE	1.4	0.7	0.3	0.339	2.413	
	HC	1.3	0.7	0.3	0.676	1.932	
IL1B	CE	1.2	0.41	0.1	0.802	1.503	0.75
	SE	1.2	0.7	0.4	0.105	2.373	
	HC	1.0	0.4	0.1	0.676	1.378	
CXCL8	CE	1.3	0.4	0.2	0.929	1.752	0.60
	SE	1.3	0.5	0.21	0.487	2.045	
	HC	1.1	0.4	0.2	0.703	1.439	
MUC4	CE	0.8	0.6	0.2	0.295	1.339	0.40
	SE	1.1	0.3	0.2	0.568	1.661	
	HC	0.7	0.5	0.2	0.220	1.162	
MUC16	CE	0.6	0.3	0.1	0.339	0.877	0.94
	SE	0.6	0.2	0.1	0.280	0.842	
	HC	0.6	0.2	0.1	0.426	0.739	

Mean, standard deviation (SD), and standard error of the mean (SEM) of CV. Lower and upper bound of confidence interval (CI) and P-value of multiple comparison (Scheffe test).

*PTGS2, prostaglandin-endoperoxide synthase 2; PTPRC, protein tyrosine phosphatase receptor type C; CEACAM1, carcinoembryonic antigen related cell adhesion molecule 1; IL1A, interleukin 1 alpha; IL1B, interleukin 1 beta; CXCL8, C-X-C motif chemokine ligand 8; MUC4, mucin 4; MUC16, mucin 16*.

### Endometrial mRNA Expression Related to Uterine Health Status

Median mRNA expression of *IL1A, IL1B*, and *CXCL8* was increased in cows with SE and CE compared with HC cows, whereas *MUC16* mRNA expression was only increased in SE cows compared with HC cows (*P* < 0.05; Figure 1). Expression of *IL1A, IL1B*, and *CXCL8* mRNA in SE was 27-, 17-, and 30-fold higher than in HC cows. Expression of *MUC16* mRNA was 2.6-fold higher in SE compared with HC cows. No differences of median mRNA expression between groups were found for *PTPRC, CEACAM1, PTGS2*, and *MUC4* (*P* > 0.05).

## Discussion

In the course of an inflammatory process, cells of the immune system synthesize a broad range of pro-inflammatory factors. The mRNA expression of certain cytokines and glycoproteins are dependent on the health status of the bovine endometrium, the stage of the estrus cycle and anatomical sections of the uterus (15–17). This study aimed to provide new information about the inflammatory response at different sites of the uterus and for cows with different uterine health status at the end of the postpartum period, i.e., when the regeneration of the epithelium is supposed to be completed. Our hypothesis was that on a molecular level, clinical endometritis is characterized by inflammatory responses spread over the entire endometrium, whereas subclinical endometritis is described by an inflammation of local regions of the uterus. The results of the multi-site comparison between the mRNA expressions of the selected transcripts, however, did not support our hypothesis since the variation of mRNA expression was independent from uterine health status. In this study, we analyzed a limited number of factors. Our selection was based on previous studies and represented factors associated with uterine disorders. In further research, the inflammatory response on molecular level should be investigated including more transcripts, additional signs of uterine inflammation as well as cow-specific factors, such as stage of lactation, parity, and the previously pregnant and non-pregnant uterine horns.

The mRNA expression at different uterine sites has hardly been investigated. Gabler et al. (16) reported first information showing no region-dependent mRNA expression for different PGE synthases and ILs comparing the *corpus uteri*, the ipsilateral, and contralateral horn from uterine tissues collected at the abattoir. Bauersachs et al. (17) identified a couple of genes with lower expression in the *corpus uteri* of healthy heifers after slaughtering, but the majority of transcripts were similar expressed across the uterine horns. In our study, mRNA expression was not different, but overall, the results indicate a high variation of mRNA expression of selected factors at the five different sites. Interleukin 1 beta is contributing to inflammatory processes by stimulating the production of *CXCL5* and by activating vascular endothelial cells to promote migration of neutrophils (20, 21). C-X-C motif chemokine ligand 8 is responsible for recruitment of PMN (22), which may explain the higher abundance in endometric cows.

In agreement with other studies (15, 23, 24), the mRNA expression of *IL1A, IL1B*, and *CXCL8* was increased in cows showing signs of CE and SE compared with healthy control cows. These results indicate the immune-modulating response of *IL*s to infections. In the present study, the mRNA expression of *MUC16* was more abundant in SE compared with healthy cows. In contrast, Wagener et al. (25) found higher *MUC16* mRNA expression in the follicular phase of subfertile and healthy cows. Mucins are protective glycoproteins for epithelial tissues against bacterial invasion (26), therefore, an upregulation in endometric cows with SE can be expected.

This study provides some new aspects of inflammatory reactions in the bovine endometrium on a molecular level and contributes to our understanding of uterine pathologies. In addition, multi-site sampling and analysis of pro-inflammatory factors may be used to improve or validate the diagnosis of endometritis.

## Data Availability Statement

The raw data supporting the conclusions of this article will be made available by the authors, without undue reservation.

## Ethics Statement

The animal study was reviewed and approved by Ethik- und Tierschutzkommission, University of Veterinary Medicine, Vienna, Austria. Written informed consent was obtained from the owners for the participation of their animals in this study.

## Author Contributions

HP: first author, contributed in sampling and writing the manuscript. PF: Contributed in laboratory work. AT: mastermind of statistical analyses, contributed to Materials and Methods. CG: supervisor of laboratory work, contributed to study design and Materials and Methods. MD: last author, mastermind of study design, supervising the manuscript. All authors contributed to the article and approved the submitted version.

## Conflict of Interest

The authors declare that the research was conducted in the absence of any commercial or financial relationships that could be construed as a potential conflict of interest.

## Supplementary Material

Supplementary Table 1 Gene transcript, primer sequences and annealing temperature used for real-time PCR with subsequently resulting amplicon length.
