# Supplementary material for: Messenger RNA Expression of Selected Factors at Different Sites of the Bovine Endometrium Associated With Uterine Health
Source: Front Vet Sci. 2021 Mar 5;8:649758. doi: 10.3389/fvets.2021.649758 (PMC7973100; doi:10.3389/fvets.2021.649758)
Supplement: Supplementary Table 1 — Gene transcript, primer sequences and annealing temperature used for real-time PCR with subsequently resulting amplicon length. [file Table_1.docx]

**Supplement Table 1**: Gene transcript, primer sequences and annealing temperature used for real-time PCR with subsequently resulting amplicon length.

| Gene | Nucleotide sequence | Amplicon length (bp) | Annealing temp. (°C) | Reference |
| --- | --- | --- | --- | --- |
| *PTPRC* | F: 5’-CAA ATT TAA ATG TGA TGG CGG-3’  R: 5’-TCG TCC ACC TGG AGT AAT CC-3’ | 286 bp | 56 | ^1^ |
| *CEACAM1* | F: 5‘-CCC AGA ACA CCT CCT ACA TG 3‘  R: 5‘-TCT GTG CAA GGA GGA GAC TC 3‘ | 336 | 58 | AY345130  540-875 |
| *IL1A* | F: 5'-TCA TCC ACC AGG AAT GCA TC-3'  R: 5'-AGC CAT GCT TTT CCC AGA AG-3' | 300 | 59 | ^2^ |
| *IL1B* | F: 5’-CAA GGA GAG GAA AGA GAC A-3’  R: 5’-TGA GAA GTG CTG ATG TAC CA-3’ | 236 | 56 | ^3^ |
| *CXCL8* | F: 5'-CGA TGC CAA TGC ATA AAA AC-3'  R: 5'-CTT TTC CTT GGG GTT TAG GC-3' | 153 | 56 | ^4^ |
| *PTGS2* | F: 5'-CTC TTC CTC CTG TGC CTG AT-3'  R: 5'-CTG AGT ATC TTT GAC TGT GGG AG-3' | 359 | 60 | ^5^ |
| *MUC4* | F: 5´-ACG TCA CTG TGC ATC TTT GG-3´  R: 5´-AAG CTC TTG ATG GAC GGT TG-3´ | 199 | 60 | ^6^ |
| *MUC16* | F: 5´-CAG GTC TCA AAA TCC CAT CC-3´  R: 5´-TGC TGG AGG TGT TGA TAT GG-3´ | 256 | 62 | ^6^ |
| *SDHA* | F: 5´-GGG AGG ACT TCA AGG AGA GG-3´  R: 5´-CTC CTC AGT AGG AGC GGA TG-3´ | 219 | 60 | ^~~7~~^ |
| *GAPDH* | F: 5’-GAA GGT GAA GGT CGG AGT CAA C-3’  R: 5’-CAG AGT TAA AAG CAG CCC TGG T -3’ | 306 | 62 | ^~~7~~^ |

*PTPRC*: protein tyrosine phosphatase receptor type C; *CEACAM1*: carcinoembryonic antigen related cell adhesion molecule 1; *IL*: interleukin; *CXCL8*: C-X-C motif chemokine ligand 8; *PTGS2*: prostaglandin-endoperoxide synthase 2; *MUC*, mucin; *SDHA*: succinate dehydrogenase complex flavoprotein subunit A; *GAPDH*: glyceraldehyde-3-phosphate dehydrogenase

1. **References**

1. Peter, S., Gärtner, M. A., Michel, G., Ibrahim, M., Klopfleisch, R., Lübke-Becker, A. et al. (2018). Influence of intrauterine administration of lactobacillus buchneri on reproductive performance and pro-inflammatory endometrial mRNA expression of cows with subclinical endometritis. Sci Rep-UK 8, 1-13.

2. Gabler, C., Drillich, M., Fischer, C., Holder, C., Heuwieser, W., Einspanier, R. (2009). Endometrial expression of selected transcripts involved in prostaglandin synthesis in cows with endometritis. Theriogenology 71, 993-1004.

3. Konnai, S., Usui, T., Ohashi, K., Onuma, M. (2003). The rapid quantitative analysis of bovine cytokine genes by real-time RT-PCR. Vet Microbiol 94, 283-294.

4. Fischer, C., Drillich, M., Odau, S., Heuwieser, W., Einspanier, R., Gabler, C. (2010). Selected pro-inflammatory factor transcripts in bovine endometrial epithelial cells are regulated during the oestrous cycle and elevated in case of subclinical or clinical endometritis. Reprod Fert Develop 22, 818-829.

5. Odau, S., Gabler, C., Holder, C., Einspanier, R. (2006). Differential expression of cyclooxygenase 1 and cyclooxygenase 2 in the bovine oviduct. J Endocrinol 191, 263-274.

6. Danesh Mesgaran, S., Sharbati, J., Einspanier, R., Gabler, C. (2016). mRNA expression pattern of selected candidate genes differs in bovine oviductal epithelial cells in vitro compared with the in vivo state and during cell culture passages. Reprod Biol Endocrin 14, 1-18.

7. Gärtner, M. A., Bondzio, A., Braun, N., Jung, M., Einspanier, R., Gabler, C. (2015). Detection and characterisation of Lactobacillus spp. in the bovine uterus and their influence on bovine endometrial epithelial cells in vitro. Plos One 10, 1-20.
